# 我如何诊断和治疗弥漫大B细胞淋巴瘤

**DOI:** 10.3760/cma.j.issn.0253-2727.2021.12.003

**Published:** 2021-12

**Authors:** 维莅 赵, 慕晨 张, 迪 付

**Affiliations:** 上海血液学研究所，医学基因组学国家重点实验室，国家转化医学中心（上海），上海交通大学医学院附属瑞金医院 200025 Shanghai Institute of Hematology, State Key Laboratory of Medical Genomics, National Research Center for Translational Medicine, Ruijin Hospital Affiliated to Shanghai Jiao Tong University School of Medicine, Shanghai 200025, China

弥漫大B细胞淋巴瘤（DLBCL）是非霍奇金淋巴瘤（NHL）中最常见的病理亚型，占所有NHL的30％～40％[1]。DLBCL具有高度异质性，不同亚型具有不同的临床特征、遗传学改变及治疗反应。R-CHOP（利妥昔单抗+环磷酰胺+阿霉素+长春新碱/长春地辛+泼尼松）是目前治疗DLBCL的标准方案，然而仍有30％～40％的患者存在耐药和复发等问题[2]。DLBCL是一种潜在可治愈性肿瘤，临床上只要条件允许应尽可能以治愈为目标，在此，我们将结合几例典型病例，对DLBCL异质性分层下的当代治疗策略进行探讨，供临床医师借鉴。

一、典型病例

例1，女，51岁。因“发现右侧腹股沟肿物2个月”就诊，淋巴结切除活检病理提示DLBCL。免疫组化示CD20、CD19、BCL6阳性，C-MYC约40％阳性，CD10、BCL2、MUM1均阴性，Ki-67 90％，原位杂交EBER（−）。FISH检测C-MYC、BCL2、BCL6重排均阴性。治疗前PET-CT可见左侧腹股沟淋巴结代谢增高，SUV_max_＝15.5，未见其他部位高代谢，骨髓检查未提示淋巴瘤累及。美国东部肿瘤协作组（ECOG）体能状态评分0分，乳酸脱氢酶（LDH）165 U/L，国际预后评分（IPI）0分。R-CHOP方案治疗4个疗程后中期PET-CT提示完全缓解（CR）（Deauville 1分），后继续利妥昔单抗单药治疗4个疗程，末期PET-CT亦提示CR（Deauville 1分），治疗结束随访至今持续缓解。

例2，男，29岁。因“发现右侧胸骨旁肿物1个月”就诊，淋巴结穿刺活检病理提示DLBCL。免疫组化示CD20、BCL6阳性，MUM1 20％，C-MYC 10％，BCL2 60％，CD10阴性，Ki-67 70％，原位杂交EBER（−）。FISH检测BCL6重排阳性，C-MYC、BCL2重排均阴性。治疗前PET-CT见全身多处骨质破坏，颈部双侧、双侧肺门、纵隔、双侧腋窝、胰腺、后腹膜、双侧腹股沟多发淋巴结肿大，胰腺多发局部高代谢，均考虑肿瘤浸润，SUV_max_＝19.3。骨髓检查提示2.19％淋巴瘤累及，ECOG 1分，LDH 326 U/L，IPI 3分，年龄调整的IPI（aaIPI）2分。R-CEOP90方案（90 mg/m²表柔比星）化疗6个疗程，中期及末期PET-CT均提示CR（Deauville 1分），骨髓微小残留病（MRD）阴性。BEAM方案预处理后行自体造血干细胞移植（auto-HSCT），治疗结束随访至今持续缓解。

例3，男，68岁。因“发现右上臂肿物1个月”就诊，切除活检病理提示DLBCL。免疫组化示CD20、CD19、CD22、CD5、MUM1、BCL6阳性，BCL2>90％，C-MYC约70％，CD10阴性，Ki-67 90％，原位杂交EBER（−）。FISH检测C-MYC、BCL2、BCL6重排均阴性。二代测序示MYD88、CD79B、PIM1、BTG1、IGLL5、ZNF608、TBL1XR1、FAS基因突变。治疗前PET-CT见右侧颌下腺内、右侧上臂皮下、纵隔、心包旁、心膈角区、右侧内乳淋巴、肝门区、后腹膜、髂血管旁、右侧腹股沟区多发淋巴结肿大，肝脏多发高代谢灶，左侧股骨骨髓腔内占位，均考虑肿瘤浸润，SUV_max_＝17.4，骨髓检查提示0.22％淋巴瘤累及，ECOG 1分，LDH 1814 U/L，IPI 4分。R-CHOP方案化疗1个疗程，二代测序结果回报后第2～6个疗程加用伊布替尼，第2～5个疗程后行腰椎穿刺+鞘内注射，中期及末期评估均提示CR（Deauville 1分），骨髓MRD阴性，治疗结束随访至今持续缓解。

二、DLBCL的临床异质性

目前临床上最常用DLBCL的预后评估体系仍然是经典的IPI。利妥昔单抗时代的改良IPI（R-IPI）将IPI原有4个危险分层简化为3个[3]。美国国立综合癌症网络IPI（NCCN-IPI）进一步细化年龄、LDH水平和限定结外受累部位[4]。在利妥昔单抗时代，上述三种预后模型均具备良好的预后指导意义，低危患者有较高的治愈机会，高危患者需采用更积极的治疗方法，而中危患者有待根据分子异质性进一步区分。

三、基于MICM分型的DLBCL诊断

DLBCL组织学分类的复杂性给诊断带来挑战，血液病理学家的丰富经验及密切结合患者的临床信息对于DLBCL的准确诊断和亚型分类非常关键。PET-CT和CT高度怀疑病变部位的粗针穿刺病理活检也使部分症状体征不典型DLBCL患者的诊断成为可能。对DLBCL亚型的诊断应遵循2016版WHO分类[1]。随着系统生物学技术的不断发展，淋巴瘤的诊断并不仅限于病理形态学（morphology）和免疫表型（immunophenotype），细胞遗传学（cytogenetics）、分子生物学（molecular）检测在诊断和分型中正发挥着越来越重要的作用，形成了淋巴瘤的MICM分型（表1）。MICM分型不仅有助于更好地区分DLBCL各亚型，也为基于分子学异常的个体化靶向治疗奠定了理论基础。

WHO根据基因表达谱不同，将DLBCL的“细胞起源（COO）”分为3类：生发中心B细胞样（GCB）、活化B细胞样（ABC）和第三型DLBCL（Type 3 DLBCL）[1]。GCB与ABC型DLBCL具有不同的潜在致病机制，GCB型的分子遗传学异常表现包括PI3K/AKT/mTOR、JAK/STAT通路异常，EZH2、CREBBP、KMT2D等表观遗传学基因突变，BCL2基因重排几乎只在GCB型中出现；ABC型则以NF-κB和B细胞受体（BCR）通路激活、MYD88和CD79B突变为主要特征[5]。COO是影响DLBCL预后的重要因素，即使在利妥昔单抗时代，nonGCB型患者的预后仍显著劣于GCB型[6]–[8]，不同COO亚型对联合免疫化疗的疗效差异可能与免疫基因表达特征有关。本中心对211例DLBCL患者利用Nanostring判断COO亚型，并分析其免疫基因表达特征。与GCB型相比，ABC型患者促炎基因表达显著上调（效应T细胞、干扰素γ和抗原提呈细胞），抗炎基因表达显著下调（调节T细胞、辅助T细胞2、髓样细胞、T细胞受体通路）[9]。

多个结外器官累及是影响DLBCL患者预后的重要临床指标。本中心对1960例DLBCL患者进行回顾性分析发现，MYD88突变是多个结外器官累及患者的特征性表现，与生殖系统、乳腺、皮肤、肾/肾上腺、骨骼受累相关[10]。RNAseq分析显示，多个结外器官累及与肿瘤浸润性调节T细胞上调和免疫微环境抑制有关。

**表1:** 弥漫大B细胞淋巴瘤（DLBCL）的MICM诊断

分类	具体内容
M（病理形态学）	参照WHO 2016版分类
I（免疫表型）	CD20、CD3、CD5、CD10、BCL2、BCL6、Ki-67、IRF4/MUM1、MYC、CyclinD1、CD30、CD23、PAX5、CD138、ALK、HHV8、SOX11、P53等；EBV原位杂交 COO分型：GCB、ABC、Type 3 DLBCL（Hans分型：GCB、nonGCB）
C（细胞遗传学）	利用FISH技术检测MYC、BCL2、BCL6重排； 利用核型分析技术检测染色体异常
M（分子生物学）	MCD亚型：MYD88L265P及CD79B突变为特征，MYD88依赖的NF-κB通路异常； BN2亚型：NOTCH2突变及BCL6融合为特征，BCR依赖的NF-κB通路异常； EZB亚型：EZH2突变及BCL2易位为特征，可根据MYC重排划分为MYC^+^/MYC^−^两类，表观遗传学基因异常； ST2亚型：SGK1突变及TET2突变为特征，PI3K、JAK-STAT通路异常； A53亚型：TP53突变及缺失为特征，潜在免疫逃逸； N1亚型：NOTCH1突变为特征，B细胞分化通路异常

注：COO：细胞起源；GCB：生发中心B细胞；nonGCB：非生发中心B细胞；ABC：活化B细胞；Type 3：第三型

20％～40％的DLBCL患者存在3号染色体BCL6基因重排，约30％的患者存在BCL2易位［即t（14;18）］。5％～15％的患者存在8号染色体MYC基因重排，可与BCL2和（或）BCL6重排同时发生，称为“双打击”或“三打击”淋巴瘤。2016版WHO分类中，双打击淋巴瘤被归为伴MYC、BCL2和（或）BCL6基因重排的高级别B细胞淋巴瘤[1]。R-CHOP方案治疗双打击淋巴瘤5年无进展生存（PFS）率仅为18％，5年OS率仅为27％。30％～35％的DLBCL患者表达MYC蛋白，20％～35％同时表达BCL2蛋白，但多数不携带MYC/BCL2异常，称作“双表达淋巴瘤”，MYC和BCL2过表达可通过免疫组化识别。标准R-CHOP方案治疗双表达淋巴瘤仍有不良预后，5年PFS率和OS率分别为32％和36％[11]。目前对于双打击和双表达淋巴瘤尚无有效的治疗措施。此外，有研究对363例DLBCL患者分析发现，87％有克隆性核型异常，最常受累的位点包括14q32、18q21、1q21、3q27、1q36、8q24、3p21、6q21、1p22和22q11[12]–[13]。

随着高通量技术不断发展，基于DLBCL遗传学特征的新的基因分型研究逐渐深入和成熟。2018年，Schmitz等[14]提出DLBCL基因分型：MCD亚型、BN2亚型、N1亚型、EZB亚型。其中MCD和N1主要起源于ABC，EZB主要起源于GCB，BN2在ABC、GCB、Type 3 DLBCL中均占有一定比例。该研究显示，46.6％的DLBCL患者可根据MCD、BN2、N1和EZB亚型分类。4种亚型在PFS和OS方面差异有统计学意义，BN2和EZB亚型的预后优于MCD和N1亚型，MCD或N1亚型的PFS率和OS率明显低于其他ABC亚型，EZB亚型的OS率明显低于其他GCB亚型。2020年，研究者结合3个不同来源DLBCL测序数据库更新了基因分型方法，将分子亚型增至七种：MCD亚型、BN2亚型、N1亚型、MYC重排阳性EZB亚型、MYC重排阴性EZB亚型、ST2亚型（主要为SGK1突变和TET2突变）和A53亚型（主要为TP53突变或缺失），其中ST2亚型具有较好的预后，A53亚型患者预后较差[15]。

本中心对349例DLBCL患者进行上述基因分型发现，仅39％的患者可根据MCD、BN2、N1、EZB、A53及ST2亚型分类。可分型的患者中，EZB及A53亚型占比较低，BN2亚型占比较高。因此，对于中国DLBCL患者同样需要一种较为简单且具有临床操作可行性的分子分型方法。我们将样本量扩大至754例，211例患者同时进行全基因组测序和PNA测序，在其中的185例中观察到55个基因突变，基于Gene Ontology数据库将突变的基因分为染色质重构、免疫应答、细胞周期/p53以及肿瘤相关信号通路BCR/NF-κB、JAK-STAT、PI3K-AKT和Wnt。根据COO分型，GCB亚型中GNA13、SGK1、SOCS1和TET2基因突变比例显著增加，染色质重塑相关基因的突变更为常见；而ABC亚型中MYD88、PIM1、DTX1和CD79B基因突变的比例显著增加，与BCR/NF-κB通路相关的基因突变在ABC亚型中更为常见。此外，MYC/BCL2双表达淋巴瘤中PIM1、BTG1和CCND3基因以及与BCR/NF-κB通路相关的基因突变比例显著增加。

四、基于异质性分层的DLBCL个体化治疗

在R-CHOP方案的基础上增加利妥昔单抗的治疗次数是提高疗效的备选方案之一。本中心对2002年至2012年间901例初治DLBCL患者进行分析，737例拟接受6个疗程R-CHOP方案化疗，其中534例完成6个疗程化疗并获得CR，根据患者意愿，264例继续接受2个疗程利妥昔单抗追加治疗。研究发现，IPI低危男性、R-IPI低危、NCCN-IPI低危、非MYC/BCL2双表达、BCR信号通路相关基因突变阳性患者可从利妥昔单抗的追加治疗中获益[16]。另一方面，国际多中心Ⅲ期GOYA研究数据显示，与6个疗程R-CHOP+2个疗程利妥昔单抗组相比，8个疗程R-CHOP方案组未观察到PFS和OS获益，调整基线差异（包括COO和IPI）后仍得到同样结果；接受8个疗程CHOP方案的患者发生3～5级不良事件和任何等级感染的风险更高[17]。中国血液/肿瘤临床多中心研究协作组（M-HOPES）发起的多中心Ⅲ期NHL001研究显示，R-CEOP70（70 mg/m^2^表柔比星）方案与R-CHOP50（50 mg/m^2^多柔比星）方案疗效相当，并且减少心脏毒性的发生，年轻、中危组患者采用蒽环类加量的化疗方案R-CEOP90方案可获益[18]。因此，我们提出对于DLBCL，应以6个疗程R-CHOP+2个疗程利妥昔单抗为经典框架，根据年龄、预后评分、剂量增加方案的可行性以及分子遗传学特征进行分层治疗。

1. 低危患者的治疗：根据FLYER研究结果，在经过严格选择的年轻低危［aaIPI＝0，不伴大包块（肿瘤最大直径<7.5 cm）］初治DLBCL患者中，4个疗程R-CHOP+2个疗程利妥昔单抗方案组与6个疗程R-CHOP方案组相比，PFS、OS、无事件生存（EFS）期、复发率的差异均无统计学意义[19]。对于低危非大包块DLBCL患者（14～75岁，IPI 0～1分），本中心正在开展Ⅲ期、随机对照NHL006临床研究（NCT02752815），4个疗程R-CHOP方案化疗并获得CR后，随机完成2个疗程R-CHOP方案+2个疗程利妥昔单抗或4个疗程利妥昔单抗。例1是年轻低危的初治DLBCL患者，使用4个疗程R-CHOP方案+4个疗程利妥昔单抗方案获得CR，预后良好，长期随访无复发。

2. 高危或中高危患者的治疗：对于年轻高危或中高危患者目前尚无标准一线治疗方案，欧洲肿瘤内科学会（ESMO）和中国临床肿瘤学会（CSCO）指南均推荐首选临床试验[20]–[21]。与R-CHOP方案相比，R-DA-EPOCH（利妥昔单抗、依托泊苷、泼尼松、长春新碱、环磷酰胺）方案可能改善IPI评分3～5分患者的生存[22]，尚缺乏R-DA-EPOCH与R-CEOP90方案的前瞻性随机对照研究结果。目前，已有4项研究对比了免疫化疗或免疫化疗后联合一线auto-HSCT治疗DLBCL的结果：有2项研究结果显示一线auto-HSCT能够改善中高危及高危组DLBCL患者的PFS[23]–[24]；另外2项研究则未显示一线auto-HSCT较剂量/密度增强的免疫化疗获得生存优势[25]–[26]。在免疫化疗时代，一线auto-HSCT治疗DLBCL的地位尚存在争议，特别是对于接受增强免疫化疗诱导治疗的患者，2018年《造血干细胞移植治疗淋巴瘤中国专家共识》建议auto-HSCT用于年轻高危DLBCL的一线巩固治疗[27]。目前M-HOPES正在开展对比R-DA-EPOCH与R-CEOP90方案联合auto-HSCT治疗年轻高危DLBCL患者的NHL005研究（NCT03213977）。上述例2为年轻中高危患者，经R-CEOP90诱导化疗后行auto-HSCT，治疗反应良好，长期随访无复发。

3. 靶向药物的应用前景：近年来，新的靶向治疗药物不断涌现，如免疫调节剂（来那度胺）、布鲁顿酪氨酸激酶（BTK）抑制剂（伊布替尼、泽布替尼、奥布替尼）、BCL2抑制剂（维奈克拉）、表观遗传药物（西达本胺、地西他滨、阿扎胞苷）、PD-1单抗等。如何有效地通过MICM分型指导靶向药物的选择，联合化疗提高患者的反应率，延长不适宜移植患者的生存是当代研究的热点话题。

比较来那度胺联合R-CHOP方案与R-CHOP方案治疗初治ABC型DLBCL患者的随机对照Ⅲ期ROBUST研究显示，两组PFS的差异无统计学意义[28]。另一项对比伊布替尼联合R-CHOP方案与R-CHOP方案治疗初治nonGCB型DLBCL的随机对照Ⅲ期PHOENIX研究显示，尽管<60岁的年轻患者可有生存获益，伊布替尼未能改善nonGCB或ABC亚型患者的EFS[29]。由此我们推测，COO分型不能准确地指导来那度胺、伊布替尼等靶向药物的应用，需更深入理解DLBCL的基因分型和分子生物学特性。如MCD亚型患者对BTK抑制剂敏感性较高，针对该亚型患者可应用BTK抑制剂联合R-CHOP方案治疗。例3为老年高危nonGCB型患者，评估其不宜接受大剂量化疗和auto-HSCT，结合其存在MYD88、CD79B、PIM1等基因突变，NCI/NIH分型为MCD型，因此我们予伊布替尼联合R-CHOP方案化疗，特别需要注意的是，老年患者应用BTK抑制剂过程中需加强对不良反应的管理，有效预防感染发生。

MYC异常是DLBCL的不良预后因素。一项Ⅱ期研究显示，来那度胺联合R-CHOP（R2-CHOP）方案可提高MYC重排阳性DLBCL患者的生存，双打击/三打击淋巴瘤患者也有66％获得CR[30]。对于这部分患者，R2-CHOP方案可使更多患者有机会获得缓解并进入后续的移植流程，期待未来行Ⅲ期对照研究证实其有效性。MYC/BCL2双表达淋巴瘤目前亦缺乏前瞻性随机对照研究，但已有R-CHOP+X的临床研究针对双表达淋巴瘤进行了亚组分析。如伊布替尼联合R-CHOP可能对MYC/BCL2高表达DLBCL患者有效[31]。BCL2抑制剂维奈克拉联合R-CHOP治疗双表达淋巴瘤，CR率可达87.5％[32]。西达本胺是我国自主研发的新一代酰胺类组蛋白去乙酰化酶抑制剂，本中心前期的一项西达本胺联合R-CHOP方案治疗老年高危DLBCL患者的Ⅱ期研究显示，双表达淋巴瘤患者的CR率为100％，2年PFS率和OS率分别为83％和91％，与R-CHOP方案历史数据比较，显著提高了CR率、PFS率及OS率[33]。基于上述结果，我们设计了西达本胺联合R-CHOP方案治疗双表达淋巴瘤的Ⅲ期研究NHL009，目前该项临床研究正在进行中（NCT04231448）。

TP53突变在DLBCL患者中的发生率为20％～25％，GCB与ABC亚型中均可检测到TP53突变，且突变率相仿。TP53突变是独立于COO、MYC/BCL-2/BCL-6蛋白过表达或基因重排的不良预后因素。增加化疗周期也不能改善TP53突变患者的生存[34]。本中心前期地西他滨联合R-CHOP方案治疗高危DLBCL患者的小样本研究显示，5例TP53突变型患者均获得CR，目前中位随访3年，5例患者均为持续CR状态[35]。

帕博利珠单抗联合R-CHOP方案治疗初治DLBCL和滤泡性淋巴瘤3B级的Ⅰ期研究疗效良好，CR率和ORR分别为77％和90％，且安全可耐受[36]。对于PD-L1高表达的患者，联合PD-1的方案可能会取得较好的疗效。

五、复发难治DLBCL患者的挽救治疗

1. auto-HSCT：在利妥昔单抗前时代，PARMA研究确立了auto-HSCT作为化疗敏感性复发NHL的挽救后巩固治疗的标准治疗地位[37]。在利妥昔单抗时代，CORAL研究显示，R-ICE方案与R-DHAP方案作为DLBCL挽救治疗方案疗效相似，总体上接受移植的患者3年PFS率为53％[38]。根据2018年《造血干细胞移植治疗淋巴瘤中国专家共识》，建议auto-HSCT用于挽救治疗敏感的复发/难治DLBCL（R/R DLBCL）的巩固治疗[27]。在临床实践中，难治性DLBCL的定义尚不明确。本中心对国内8个中心2342例患者进行队列分析，探索难治性DLBCL的定义，发现免疫化疗无缓解或在auto-HSCT后1年内复发能够筛选出预后不良的患者，适合用于定义难治性DLBCL[39]。在此定义下，难治DLBCL的5年累积发病率为20％，后续挽救化疗的ORR仅30％，CR率仅9％。由于挽救化疗的疗效不佳，患者总体生存情况差，中位生存时间仅5.9个月，2年OS率仅16％。

2. CAR-T细胞治疗：在已发表的各项研究中，CAR-T细胞疗法均显示出良好的疗效，有效率达70％～80％，CR率均为50％左右[40]–[42]。CAR-T细胞疗法的最大优势在于无论患者对化疗是否敏感，均可获得疗效。目前，抗CD19 CAR-T细胞疗法阿基伦赛注射液和瑞基奥仑赛注射液已被我国NMPA批准用于二线治疗失败的R/R DLBCL。本中心前期研究报道了CD19 CAR-T细胞治疗产品JWCAR029治疗复发B-NHL的疗效，ORR为100％，其中CR率为66.7％[43]。研究发现，CAR-T细胞治疗的疗效与淋巴瘤微环境的免疫抑制状态有关，免疫抑制状态越强，肿瘤相关巨噬细胞激活越明显，患者越难在CAR-T细胞治疗后达到CR。同时研究发现部分免疫检查点与患者的疗效存在关联，对于CAR-T细胞治疗可能无效的患者，使用CAR-T细胞治疗联合免疫检查点抑制剂可能提升患者的疗效。

3. 异基因造血干细胞移植（allo-HSCT）：allo-HSCT也可治愈R/R DLBCL患者，但较高的移植相关死亡率通常会抵消其移植物抗肿瘤效应的优势，因此只适用于少部分经过严格筛选的患者。根据2021年NCCN指南，对于既往接受二线及二线以上治疗的复发患者，在治疗后获得高质量CR/PR后可能获益于allo-HSCT，因此，对于这部分患者应考虑行allo-HSCT。此外，对于其他患者，如果没有条件开展CAR-T细胞治疗，对符合条件的患者也可行allo-HSCT。

综上所述，DLBCL是一类临床表现和生物学行为呈高度异质性的肿瘤，应采用基于临床、分子异质性分层的个体化治疗，对于低危患者，在8个疗程利妥昔单抗的基础上，可以在保证疗效的情况下进行化疗简化。对于中危患者，增加蒽环类药物可以带来获益，需要其他临床研究确定是否所有中危患者在疾病缓解后均需进行auto-HSCT。对于高危、适合移植的患者，大剂量化疗序贯auto-HSCT是首选治疗，对于不适宜移植的患者，我们期待进一步通过分子机制的转化研究，即基于MICM分型判断患者预后和选择治疗药物，最终实现DLBCL从经验性治疗到智慧化治疗的策略转变，使每例患者拥有治愈的可能。
